# The Impact of Food Additives on the Abundance and Composition of Gut Microbiota

**DOI:** 10.3390/molecules28020631

**Published:** 2023-01-07

**Authors:** Xuewei Zhou, Kaina Qiao, Huimin Wu, Yuyu Zhang

**Affiliations:** 1 Food Laboratory of Zhongyuan, Beijing Technology and Business University, Beijing 100048, China; 2 Key Laboratory of Flavor Science of China General Chamber of Commerce, Beijing Technology and Business University, Beijing 100048, China

**Keywords:** gut microbiota, food additives, preservatives, antioxidants, sweeteners

## Abstract

The gut microbiota has been confirmed as an important part in human health, and is even take as an ‘organ’. The interaction between the gut microbiota and host intestinal environment plays a key role in digestion, metabolism, immunity, inflammation, and diseases. The dietary component is a major factor that affects the composition and function of gut microbiota. Food additives have been widely used to improve the color, taste, aroma, texture, and nutritional quality of processed food. The increasing variety and quantity of processed food in diets lead to increased frequency and dose of food additives exposure, especially artificial food additives, which has become a concern of consumers. There are studies focusing on the impact of food additives on the gut microbiota, as long-term exposure to food additives could induce changes in the microbes, and the gut microbiota is related to human health and disease. Therefore, the aim of this review is to summarize the interaction between the gut microbiota and food additives.

## 1. Introduction

Food additives are an important part of modern food industry, as they are used in food to maintain or promote its taste, safety, freshness, appearance, and nutritional quality. There are 22 categories and more than 2000 kinds of additives permitted in China [1], whereas this number in European Union and United States is more than 1500 and 4000, respectively [2,3]. The regulations and rules set by different government organizations are not totally the same. The regulations and rules of food additives used in food are authorized in the Code of Federal Regulations Title 21 set by United States Food and Drug Administration. And a new food additive application must include the basic physicochemical properties and provide the information about safety assessment in human health [2]. For the European Union, food additives are administrated by European Food Safety Authority (EFSA) and the Regulation (EC) No. 1333/2008 of the European Parliament and of the Council [3]. In Canada, the additives used in food are administrated by the List of Permitted Food Additives and Marketing Authorization, whereas in Australia and New Zealand, the Australia New Zealand Food Standard Code—Standard 1.3.1—Food Additives regulates the additives that permitted using in food [2]. All those regulations and rules have limited the food categories and dose which the additives can be used. The category of food additives mainly includes flavor enhancers, antioxidants, preserves, colorants, and sweeteners. The food additives are obtained from natural substances extraction or artificial synthesis. The artificial food additives are widely used in processed food due to their low cost, better stability, and uniformity [4]. In the French market, 53.8% (N = 126, 556) of food products contained at least 1 food additive and 11.3% at least 5 food additives [5]. The safety and toxic characteristics must be evaluated systematically before those additives are used in food, including through in vivo and in vitro trial [6,7,8]. There are studies focusing on the safety qualities of additives used in food, such as artificial sweeteners; the risk of cardiovascular diseases; and the metabolization of Allura red in vivo [9,10]. Azo dyes, including Sunset Yellow, tartrazine, Allura Red, and amaranth, have been reported with developmental toxicity potential in zebrafish embryos [11]. The EFSA Panel on Food Additives and Flavourings [12] has revaluated the safety of phosphoric acid-phosphates as food additive. In an in vitro study, butylated hydroxytoluene (BHT) induced non-apoptotic cell death in rat thymocytes [13] and can potential in enhancing ELT-3 rat leiomyoma cell proliferation [14]. However, most researches focus on the safety properties of artificial food additives, and there have been fewer studies on the effect on gut microbiota characteristics induced by food additives used in processed food. As the function of gut microbiota on host gut health, metabolism, immune, inflammation, and diseases has been studied deeply [15,16,17,18], researchers have raised concerns about the effect of food additives on the gut microbiota. Food additives can affect the gut microbiota such as *Firmicutes*, *Bacteroidetes*, *Barnesiella*, *Prevotella*, *Ruminococcaceae*, *Bifidobacterium*, etc. [19], and those microbiomes were reported to maintain gut barrier, participate in short chain fatty acid (SCFAs) metabolism as well as decomposition of fiber and sugar, and are associated with obesity and diabetes. The question is whether the artificial food additives affect the health and diseases of the host by regulating the gut microbiota.

This project is focused on artificial food additives, including antioxidants, preservatives, sweeteners, flavor enhancers, and colorants. The keyword additives (flavor enhancer, antioxidants, preservative, artificial colorants, and sweeteners), gut microbiota (or intestinal microbiota, or gut flora) were searched in the title, abstract, and keywords that were published in past ten years on the websites Web of Science, ScienceDirect, and PubMed. This review was aimed at the impact of artificial food additives on gut microbiota.

## 2. Gut Microbiota

### 2.1. The Function of Gut Microbiota

The gut microbiota is the sum of different kinds of microbes in the gastrointestinal tract, which influence the host’s health through the ability of digestion, metabolism, and excretion. The main phyla of gut microbiota are *Bacteroidetes*, *firmicutes*, *fusobacteria*, *proteobacteria*, *cyanobacteria*, *verrucomicrobia*, and *actinobacteria* [20]. The gut microbiota functioned in human health has been reported to be associated with the digestion and adsorption of nutrients through fermentation and decomposition of nutrients in the colony [21,22]. The glycoside hydrolase gene representation in gut microbiota is associated with the degradation of fiber and production of SCFAs [23]. The relative abundance of *Ruminococcus 2*, *Faecalibacterium*, and *Akkermansia* was associated with increased neutrophil rates and the total bacterial abundances of *Faecalibacterium* was associated with neutrophil dynamics, which revealed the function of gut microbiota in the immune system [24]. In the gut-brain axis, the modification of gut microbiota can promote endocrinal cells to release neuropeptides, peptides, and enteric neuroendocrinal factors [25]. Meanwhile, in the gut-liver axis, the proinflammatory markers, iNOS and MMP-2, that released by gut microbiota are enriched due to the alteration of the gut microbe’s environment [26]. The gut microbiota also acts as biomarkers in diseases, such as gout and lung cancer [27,28]. Furthermore, a cohort study in an obese population found that the relative abundance of *Prevotella* was correlated with Body Mass Index (BMI) [29]. The gut microbiota is also part of the intestinal barrier and contributes to mucosal immunity [30,31]. The microbiota and its metabolites largely impact the intestinal barrier integrity, host metabolism, and pathogen colonization [16]. Fermentation of carbohydrates is a core function of the gut microbiota [32]. In the colon and cecum, the dietary fiber and some resistant starches are fermented by gut microbiota, such as *Bifidum*, *Bifidobacterium*, *Butyrivibrio*, and generated SCFAs [33]. The SCFAs mainly include acetate, propionate, butyrate, and pentanoate, which serve as signaling molecules that activate the G-protein-coupled receptors (GPCRs) and contribute to the formation of an anti-inflammatory environment in the intestinal trial [34]. Vernocchi et al. [28] have found a strong correlation between the presence of SCFAs and the normal healthy gut microbiome. Moreover, the microbial metabolite, urolithin A, derived from microbial transformation, displays serveries activities, including anti-inflammatory, antioxidative, and anti-ageing [35]. The physiologic processes of lipid digestion and absorption are central functions to the jejunum [36]. The bile acids can facilitate the digestion and adsorption of lipids after secretion into the small intestine, and a small portion that enters the colon can return to the liver after microbial biotransformation [37]. In addition, the function of gut microbiota-derived vitamins plays an important role in psychiatric health and disease [38]. Instead, the gut microbiota was associated with diseases regulation, such as type 2 diabetes and obesity. The interaction between the gut microbiota and the regulatory system have been considered to affect human neurophysiology and mental health [39]. Therefore, the microbiota and its metabolites play an important role in host physiology and pathology.

### 2.2. Effect of Dietary Component on Gut Microbiota

Intestinal microbiomes use dietary components as substrates for fermentation, and the produced metabolites participate in various physiological processes. Normally, the gastrointestinal microbiota is stable and maintains balance in homeostasis with its host [40]. The gut microbiota changes rapidly after dietary ingestion and responds rapidly to the altered diet, those features potentially facilitating the diversity of human dietary lifestyle [41]. The type and component of the food, non-nutritional materials, or the toxic substance that are ingested into the gastrointestinal system can all induce structure and abundance alternation of the gut microbiota in the intestine [23,42]. Therefore, metabolites generated by intestinal microbiota using dietary nutrients as fermentation substrates will be changed [43]. Dietary protein, including protein source [22,44], processing factors [45], and protein oxidation, can affect the dietary protein digestibility on gut microbiota by influencing fermentation, adsorption, and functional properties of protein in the intestine, and finally, can impact the composition of the gut microbiota and thus affect human health [46]. A randomized controlled-feeding trial with overweight adults has found that a fried meat diet lowered the richness of the microbial community and decreased the abundance of *Lachnospiraceae* and *Flavonifractor*, accordingly, the lower concentration of butyric acid, valeric acid, and 3-indolepropionic acid, and higher concentration of methylglutaric acid were observed; those metabolites were associated with inflammation levels [45]. *Lachnospiraceae* can prevent obesity, and the abundance of *Flavonifractor* is negatively correlated with obesity [45]. The stewed pork protein diet changed the production of SCFAs by increasing *Sphingomonadaceae*, while decreasing *Eggerthellaceae* and *Lactobacillaceae* [22]. Rats fed with hen egg white induced an increase in the *Akkermansia* relative abundance, while those fed with duck egg white observed a higher relative abundance of *Peptostreptococcaceae* and *Proteobacteria*, and decreased in the relative abundance of *Lachnospiraceae* [47]. Considering the function of those microbes in the intestine, *Akkermansia* were shown to be responsible for increased intestinal permeability and facilitating pathogen entry [48]. *Peptostreptococcaceae* has the potential to maintain intestinal homeostasis [47]. *Proteobacteria* can synthesize both pantothenic acid and coenzyme A (CoA) [38]. While the complex plant material that cannot be digested in the host gut, it can be decomposed by *Lachnospiraceae* [46]. Xie et al. [22] found that soybean protein was beneficial for the microbial metabolism, but may reduce the intestinal peristalsis of mice when compared with intake casein and processed meat protein. Omnivore and vegan diets (containing fiber) can alter fecal amino acid levels by promoting the growth of *Firmicutes* for amino acid metabolism [23]. The high-fiber diet increased the *Clostridiaceae* abundance in the jejunum and ileum of mice, and decreased the abundance of *Bacteroidaceae* and *Bifidobacteriaceae* in all intestinal tract [18]. *Clostridiaceae* can increase the expression of enzyme Dgat2 that is critical for lipid TG synthesis and storage both in in vitro and in vivo studies [18]. The increased abundance of genus *Lachnoclostridium* and *Lactobacillus* induced by collagen-induced arthritis (CIA) was eliminated in mice fed a high-fiber diet rich with resistant starch [49]. *Lachnoclostridium* has a known function in butyrate production [43]. *Bifidobacteriaceae* and *Lactobacillus* are two important components of the beneficial bacterial community and in cross-talk with the host through adhering to epithelial cells, colonization in gastrointestinal mucosa, defensing pathogens, and regulating the immune system [50]. Moreover, minerals and vitamins also alter the composition and diversity of gut microbiota, such as selenium [51] and vitamin E [21]. And the express genetic machinery of majority gut microbes enables them to synthesize and metabolize various vitamins, especially B vitamins [38]. Therefore, diet allows to affect the component and abundance of gut microbiota, and the gut microbes also functioned in the digestion and metabolism of dietary ingredients.

### 2.3. Gut Microbiota and Diseases

The dysbiosis of gut microbiota causes diseases or promotes the development of disease. The gut microbiota has been found with typical composition and abundance in special diseases. Microbial metabolites play an important role in developing obesity, acting as a bridge linking diet and obesity [43]. Obesity was associated with elevated levels of circulating succinate; the relative abundance of *veillonellaceae* and *prevotellaceae*, which produce succinate, was higher in obese individuals, while the abundance of *clostridaceae* and *odoribacteraceae*, which consume succinate, was lower [52]. *Faecalibacterium prausnitzii* is a marker of lower visceral fat mass, while *Clostridium CAG 58* and *Flavonifractor plautii* are the taxa that associated with obesity [53]. Gut dysbiosis was observed preceding clinic symptoms in rheumatoid arthritis (RA) and has been implicated in the initiation and persistence of RA [49]. Furthermore, the increased abundances of *Lachnospiraceae_NK4A136_group* and *Bacteroidales_S24-7_group* were observed in CIA mice [49]. Gut microbiota can be used as a sensitive biomarker for diagnosing gout, since *Bacteroides xylanisolvens* and *Bacteroides caccae* are enriched while *Bifidobacterium pseudocatenulatum* and *Faecalibacterium prausnitzii* are depleted in diseased individuals [54]. In addition, the gut microbiota promotes catabolic metabolism of purines and uric acid, and its metabolites increase uric acid excretion [55]. Compared to healthy controls, the relative abundances of *Bacteroides*, *Prevotella*, and *Fusobacterium* were increased in gout patients, whereas *Enterobacteriaceae* and butyrate-producing species were decreased [27]. The gut microbiota and its metabolites regulate the gene expression in liver, which can induce imbalance in the gut-liver axis and may cause disease, including carcinogenesis [15]. Changes in the microbiomes in the gut were found to be related with breast cancer [56]. *Enterococcus faecalis* levels were associated with variants at the MED13L locus that linked to colorectal cancer [57]. The gut microbiota in non-small cell lung cancer (NSCLC) patients was imbalanced, wherein the abundance of *Akkermansia muciniphila*, *Bacteroides*, *Rikenellaceae*, *Mogibacteriaceae*, *Clostridiaceae*, and *Peptostreptococcaceae* were found lower than controls [28]. In the event of the COVID-19 pandemic, recent research has found that gut microbiota-mediated amino acids, sugar metabolites, and neurotransmitters are involved in multiple cytokine dynamics in COVID-19 [58]. Those reveal the role of microbes in the gut-lung axis. The disordered gut microbiota will disrupt the gut immune system and induce diseases, such as inflammatory bowel diseases (IBDs), ulcerative colitis (UC), and Crohn’s disease (CD) [17]. The previous study observed that *Proteobacteria* and *Bacteroidetes* were significantly increased in CD patients when compared to healthy subjects; indeed, the diversity of Bacteroidetes was far less than Firmicutes in CD patients [59]. Zhen et al. [60] found increased relative abundance of the Bacteroidetes phylum and decreased Firmicutes phylum in the gut of ABX mice, thus leading to a disorder of SCFAs metabolism. Moreover, recognition and working memory can be influenced by *Lactobacillus acidophilus*, *Lactobacillus fermentum*, *Bifidobacteria*, *Bifidobacterium bifidum*, *Lactobacillus casei*, and *C. difficile*, through the gut-brain axis in Alzheimer’s disease [61]. Trimethylamine-N-Oxide produced by *Proteobacteria* metabolization in the gut is implicated in developing Acute Coronary Syndrome (ACS) [62]. Based on those results, the gut microbiota was proved to be associated with the host’s diseases.

## 3. Effects of Food Additives on Gut Microbiota

### 3.1. Antioxidants

The food additives in one’s diet do affect the composition and abundance of the gut microbiota in the intestine. The food additives and their association with gut microbiomes summarized in this project are presented in Table 1.

Antioxidants are a kind of food additive that can be used in foodstuff with regulated amounts to avoid oxidation of food products and improve the storage duration [84]. The antioxidants include natural antioxidants (e.g., tocopherols) and synthetic antioxidants (e.g., phenolic antioxidants); these antioxidants can prevent free radicals chain reactions of oxidation [85]. Antioxidants are commonly used in the food processing industry, especially in edible oil and fat; thus, oil and fat are widely used as materials in different kinds of processed food.

A survey about the synthetic phenolic antioxidants (SPAs) in foodstuffs from ten provinces in China found that more than 99% samples detected at least one of the SPAs, the first three common SPAs being BHT, BHT-Q, and butylated hydroxyanisole (BHA), which totally accounted for 83.2% of total SPAs contents in thirteen food categories (N = 289) [86]. Although the antioxidants were considered safe within moderate amounts, the consumers were worried about the health effect induced by antioxidants added in food [87]. An in vitro study has evaluated the susceptibility of human gut microbes to phenolic compounds. Natural phenolic compounds (such as eugenol, ferulic acid, and vanillin) decreased the growth of *Agathobacter* and *Clostridium* strains, and the *Bacteroidetes* and *Actinobacteria* strains were mostly not susceptible to phenolics [88]. However, the effect of synthetic antioxidants on the gut microbiota still needs to be studied.

### 3.2. Preservatives

Food preservatives are used to ensure safety and prevent quality loss derived from physical-chemical, microbial, or enzymatic reaction [89]. Some of the preservatives are also active as antioxidants, such as sulfur dioxide, sodium metabisulphite, sodium sulfite, and potassium sorbate [1]. In this project, synthetic preservatives were of concern, including sodium benzoate, benzoic acid, ethylparaben, sodium nitrite, nitrite, sodium sulphite, and potassium sorbate.

An in vivo study was done in pigs fed with a benzoic acid-supplemented nursery diet. The transition of the bacterial community was mainly driven by the decreased abundance of the genus of *Prevotella* and the phylum of Bacteroidetes [65]. The abundance of *Fusicatenibacter*, *Ruminococcus*, and *Escherichia-Shigella* in pigs fed with a diet containing 90% benzoic acid and 10% essential oil (include thymol, 2-meth-oxyphenol, and eugenol) were significantly (*p* < 0.05) increased compared to control (without additive), while *Prevotella*, and *Coprococcus 1* were significantly decreased [66]. In another piglet trial, 49% benzoic acid supplementation diet was observed with higher abundance of *Ruminococcus* (False Discovery Rate, FDR < 0.01), *Fibrobacteraceae* (FDR < 0.05), and *Prevotellaceae* (FDR < 0.01), bacteria which were confirmed with certain fiber fermenting abilities [67]. However, there is also research that found no significant difference of benzoic acid supplementation on pig jejunum and cecum microbial populations [90]. Meanwhile, the gut microbiota of wild-type C57BL/6 mice (male) fed with sodium benzoate-supplemented diet for 8 weeks was studied, and a significant decrease was observed in the *Coriobacteriaceae* family, which can convert carbohydrates to acetic acid and lactic acid in mice [63]. Lastly, in human volunteers, sodium benzoate promoted the growth of *Bifidobacterium* [69].

Xu et al. [68] found that both low dose nitrite (0.15 g/L) and high dose nitrite (0.30 g/L) could significantly upregulate α-diversity in C57BL/6 mice on day 120. The result of α-diversity includes the increase of Chao 1 and Shannon index, which revealed that the total number of operational taxonomic units (OTUs) is increased and the diversity is higher. In addition, the markedly different genera were higher in day 120 than in day 70. The low dose nitrite–treated mice uniquely upregulated the abundances of *Alloprevotella*, *Coprococcus*, *Acetatifactor*, and *Falsiporphyromonas*, while downregulated the abundances of *Elusimicrobium*, and *Akkermansia*. Those results revealed that long-term exposure to nitrite significantly alters the abundance of gut microbiota in C57BL/6 mice [68]. *Akkermansia* was reported as a next-generation beneficial microbe, which is negatively associated with obesity, diabetes, cardiometabolic diseases, and low-grade inflammation [91,92]. In a dextran sodium sulfate (DSS)–induced mouse model, genus level of *Prevotellaceae_UCG-001*, *Ruminococcaceae_UCG-014*, and *Lactobacillus* were increased in NaNO_3_ treated (2 mM in drinking water, 5 days) mouse; moreover, the enriched metabolic pathways of p53 signaling and colorectal cancer was partially decreased [71].

In an in vitro study, the human gut microbes were found to be highly susceptible to sodium nitrite, sodium benzoate, and potassium sorbate, especially, *Clostridium tyrobutyricum* or *Lactobacillus paracasei*, which have known anti-inflammatory properties, were significantly more susceptible to those three preservatives than *Enterococcus faecalis* or *Bacteroides thetaiotaomicron* that have known pro-inflammatory or colitogenic properties [64]. Potassium sorbate can significantly decrease the *Coriobacteriaceae* family, which can convert carbohydrates to acetic acid and lactic acid in mice [63]. Compared to control (sulfite free media), substantial decrease of *Rhamnosus*, *Lactobacillus species casei*, *Streptococcus thermophilus*, and *Plantarum* were observed in media containing concentrations of sulfites between 250 and 500 mg/L after being exposed to in vitro bacterial culture for two hours [70]. In a human volunteer’s trial, the propionic acid was found to increase while acetic acid decreased with the presence of sodium sulfite; indeed, the result of Shannon α-diversity showed that the addition of sodium sulfite increased the abundance of *Escherichia/Shigella*. In addition, sodium sulfite had an inhibitory effect on the growth of *Bifidobacterium* [69]. In wild C7BL/6 mice, ethylparaben showed significantly (*p* = 0.0424) hyperglycemic, and the relative abundance of *Proteobacteria* was enriched by ethylparaben compared to the control group [63].

### 3.3. Flavor Enhancers

Flavor enhancers are multiple substances used in food to promote taste, especially umami. Amino acids and nucleotides are flavor enhancers in common use, among which monosodium glutamate (MSG) is most widely used in processed food and is presented in this section. In addition, novel umami agents, such as protein hydrolysate and umami peptides [93,94], attract increasing attention and have the potential to become new flavor enhancers. However, the effect of flavor enhancers on gut microbiota is mainly focused on MSG, and relevant experimental data for those novel umami agents are still lacking.

The most commonly used flavor enhancer is monosodium glutamate (MSG, C_5_H_8_NO_4_Na), whose chemical structure is sodium salt from glutamic acid. Xu et al. [72] have studied the intestinal structure and the intestinal microbiota with MSG oral gavage to mice. The ratios of Bacteroidetes and Firmicutes in the 30 mg/kg (L-MSG) group were lower than those in the 300 mg/kg (M-MSG) and 1500 mg/kg (H-MSG) groups. Additionally, compared with the control group, the proteobacteria decreased in H-MSG group, but increased in M-MSG group. On the other hand, Peng et al. [73] have observed that MSG did not significantly alter the community structure and functional features of gut microbiota in human volunteers during a four-week experiment with 2 g MSG per day. Although some bacteria including *Megamonas*, *Faecalibacterium*, *Collinsella*, and *Blautia* tended to change, there was no significant difference in the alteration of all genera. At the functional level, the microbial functions were rich, mainly distributed in membrane transport, amino acid metabolism, and carbohydrate metabolism, but there was no significant difference between samples obtained at different times.

### 3.4. Sweeteners

Sweeteners are closely related to food flavor and human health, as consumers are more and more considering the health problems both certainly and potentially related to sugars. A prospective NutriNet-Santé cohort (103,388 participants) suggested that artificial sweeteners might represent a modifiable risk factor for cardiovascular disease prevention [10]. The effect of artificial sweeteners, acesulfame-K, aspartame, saccharin, sucralose, cyclamate, and neotame, on gut microbiota has been reviewed by Cao et al. [95], whereby those sweeteners could cause gut dysbiosis, which could lead to impaired glucose metabolism in rodents. Similar results were also reviewed by Ruiz-Ojeda et al. [96]. Gultekin et al. [97] have summarized that acesulfame-K, aspartame, saccharin, and sucralose are likely to destroy glucose tolerance and support weight gain by negatively affecting microbiota. Sugar alcohols are a group of polyols which are produced from sugars and are less digestible since they are difficult to totally digest in small intestine; therefore, some of them can be fermented in the colon [97]. The polyols can be used in sugar free food, since they do not induce salivation and do not interfere with the glucose levels in blood [98]. In a previous review, sugar alcohol was known to increase the number of *bifidobacteria* in the microbiomes and can induce dose-dependent flatulence in the colon [96]. Studies on the effect of sugar alcohol on the gut microbiota have been conducted within the last ten years. In this section, xylitol, sorbitol, erythritol, and lactitol are evaluated.

There are some in vivo data about the effect of xylitol on the gut microbiota in the intestine. Due to it characteristic of being less digestible in the intestine, the specific experiments on high-fat diet with xylitol supplement were evaluated in mice. Compared to the high-fat diet mice, the relative abundances of *Proteobacteria*, *Bacteroidetes*, and *Actinobacteria* were decreased, while the relative abundances of Firmicutes and ratio of *Firmicutes/Bacteroidetes* were increased in C57BL/6 mice that fed with high-fat diet supplemented with 10 g/L xylitol [74]. In addition, Uebanso et al. [75] gave a high-fat diet with 194 ± 25 mg/kg b.w. supplement of xylitol to C57BL/6J mice and found that the Bacteroidetes phylum and genus *Barnesiella* abundance were reduced, while the abundance of Firmicutes phylum and genus *Prevotella* were increased. Altered gut microbiota composition was present in the rats fed with 10% xylitol for 15 days, wherein the genera *Ruminococcaceae* and *Prevotella* was significant decreased, while *Bacteroides* was notably increased [76]. The results above showed similar changes of gut microbiota after the xylitol intake from feed. It has been reported that xylitol consumption by mice showed positive effect on the metabolic activity of a number of gut microbial populations [99]. However, in an in vitro single-phase continuous fermentation model, the gut microbiota composition was found differentiated after xylitol supplementation (1.67 g/L) only for the first 3 days; additionally, xylitol significantly enhanced the relative amount of *Clostridium* and *Phascolarctobacterium*, which act as butyrate synthesizing bacteria [77]. Meanwhile, xylitol has increased the production of butyrate and propionic acid. The same result was reported by Yue et al. [100] that xylitol produced mainly butyrate, which may play a major role in improving gut barrier function. The population sizes of Escherichia were increased beyond expectation after xylitol supplementation [77]. On the contrary, Xiang et al. [101] observed no significant of xylitol on the composition of gut microbiota both in vivo and in vitro, but observed the increasing contents of all SCFAs. This may be induced by key enzymes (xylulokinase, xylitol dehydrogenase, and xylulose phosphate isomerase) in xylitol metabolism which present in Bacteroides and *Lachnospiraceae* metabolites [101].

For long-term intake of sorbitol, Li et al. [78] found that the relative abundances of *Bifidobacterium*, *Lachnospiraceae NK4A136*, *Lachnospiraceae UCG 001*, *Candidatus Arthromitus*, *Eubacterium ventriosum*, and *Ruminococcus torques* were significantly decreased, while the relative abundances of *Tyzzerella*, *Helicobacter*, *Prevotella 9*, and *Alistipes* were increased in mice. An in vitro growth assay using no carbon-defined media with sugar alcohols supplement showed that *Clostridia* and *Erysipelotrichia* were isolated only in sorbitol as a carbon source [102]. Furthermore, Hattori et al. [79] found that the gut microbiota showed a positive impact on sorbitol-induced diarrhea; treatment with sorbitol resulted in the greatest increase at genus level of the abundance of *Klebsiella*, *Escherichia*, *Proteus*, and *Enterobacter* in the family *Enterobacteriaceae*. Those results revealed that sugar alcohols are a major carbon source for the fermentation of gut microbiota.

Erythritol (E968) was proposed as a food additive by EFSA in 2015 [103]. Ninety percent of erythritol is absorbed in the small intestine, and ten percent enters the colon, and the in vitro trial found that no consistent disruption in the α-diversity was observed in human gut community [104]. In participants (diabetic and non-diabetic patients) with lactitol administration for two weeks, the abundance of *Actinobacteria*, *Actinobacteria*, *Bifidobacteriales*, *Bifidobacteriaceae*, and *Bifidobacterium* were found with an increasing trend [80]. Moreover, an in vitro colonic fermentation study observed that fermentation of lactitol produced mainly acetate [100]. This may result in gut microbiota that metabolize SCFAs.

### 3.5. Colorants

The synthetic food colorants used by food manufacturers have been increasing due to their low cost, better stability, high color intensity, and uniformity [4]. The food safety management of government and non-government organizations have strictly defined the range and dosage of using colorants. The synthetic colorants, including tartrazine, Sunset Yellow FCF, ponceau 4R, Allura Red AC, quinoline yellow, and carmoisine, have been reported associated with hyperactivity in children [25]. Another colorant, titanium dioxide, is forbidden for use in food in the European Union [105]. However, those additives were permitted for use in specific food categories with limited doses. This section evaluates the information about artificial colorants that are used in processed food with their effect on the gut microbiota.

Tartrazine exposure induced gut microbiota dysbiosis in the juvenile crucian carp fish (*Carassius carassius*) [106]. In an in vitro trial, *Escherichia coli*, *Enterococcus faecium*, *Aerococcus viridans*, and *Bacillus cereus* can decolorize Sunset Yellow, and tartrazine after 30 min contact, which means those microbiomes have azoreductase activity [82]. In animal studies, ponceau 4R was found merely absorbed in the digestive tract, where it is anaerobically reduced by microflora, with small levels of the resulting metabolites systemically absorbed [107]. Allura Red AC has been reported to induce colitis in the context of dysregulated interleukin -23 [108]. An in vivo challenge of primed mice with Red 40 (Allura Red AC) promoted rapid activation of CD^4+^ T cells [109], while in CD^4+^ T cells, the gut microbiota-reactive interleukin -17-producing Th17 cells are central to the pathogenesis of certain types of IBD [110]. The results presented that Allura Red AC can induce inflammation of intestine by regulating the immune cell secretion. At phylum level, the proportion of *Verrucomicrobia* after oral administration of micro-TiO_2_ (10, 40, 160 mg/kg bw) was significantly lower than that in the control group (*p* < 0.05), and the proportion of Bacteroidetes at 10 mg/kg group decreased to 28.20%, while that of *Firmicutes* increased significantly to 70.23% (*p* < 0.05) [81].

### 3.6. Other Food Additives

There are several artificial food additives which are not included above, such as emulsifiers carboxymethylcellulose, polysorbate 80, resistant starch, sodium stearoyl lactylate, maltodextrin, and carboxymethyl cellulose. Those food additives are evaluated in this section.

Emulsifiers, carboxymethylcellulose, and polysorbate 80 (P80) develop dysbiosis with overgrowth of mucus-degrading bacteria, as well as further deficiency in interleukin-10 or toll-like receptor 5 [111]. However, the emulsifiers used to maintain food-specific properties may increase the translocation of pathogenic microbes in the intestinal epithelial barrier and cause the initiation of intestinal inflammation and consequently cause the increase in the incidence of inflammatory bowel disease [30]. Maltodextrin and carboxymethyl cellulose induced the decreasing of α-diversity, and both decrease in acetic acid levels, whereas the lower acetic acid levels were correlated with higher *Akkermansia* abundance and lower abundance of *Bacteroides* and *Streptococcus* [112]. The increased *Lachnoclostridium* and *Lactobacillus* genera abundance concomitant with CIA were eliminated by a resistant starch-high fat diet. Notably, resistant starch supplement also led to a predominance of Bacteroidetes, and increased the abundances of *Bacteroidales_S24-7_group* and *Lachnospiraceae_NK4A136_group* genera in CIA mice [49]. The effect of sodium stearoyl lactylate (SSL) on fecal microbiota was studied in vitro, wherein 0.025% (*w*/*v*) of SSL was found to reduce the relative abundance of the *Clostridia* class. The relative abundance of the families *Lachnospiraceae*, *Ruminococcaceae*, and *Clostridiaceae* was substantially reduced, whereas that of *Bacteroidaceae* and *Enterobacteriaceae*, *Desulfovibrionaceae* was increased. The genome reconstruction analysis found that SSL significantly reduced concentrations of butyrate and increased concentrations of propionate compared to control cultures [83].

## 4. Summary

As summarized from the literatures, food additives can induce the alteration of the gut microbiota in the host intestine. The in vivo animal model and in vitro bacteria culture were used to evaluate the effect of food additives on the gut microbiota. The association between artificial antioxidants and gut microbiota was minor; conversely, the natural antioxidants attract more attention. Preservatives highly showed association with the microbes with SCFAs generation. Monosodium glutamate presented a positive (or no difference) effect on the composition of gut microbiota. The sugar alcohol sweeteners can be used as fermentation of gut microbiota, and most polyols will alter the composition of gut microbiota. However, the definite impact of food additives on gut microbiota is not illustrated clearly, even though various studies have been reviewed. Different food additives lead to different effects on the same phylum or genus of gut microbiota. It is difficult to summarize what kind of changes will be induced by those food additives.

Even though there are several studies on the additives that affect the gut microbiota, the knowledge about the effects on gut microbiota induced by multiple artificial, especially synthetic, additives are not sufficient. The systematic studies about the effects and functions of artificial antioxidants and synthetic colorants on gut microbiota are few. Therefore, those food additives should be studied further.

## Figures and Tables

**Table 1:** The effect of food additives on gut microbiota and its function.

Food Additives	Experiment Trial	Microbes	Function	References
Sodium benzoate	C57BL/6 mice	*Coriobacteriaceae*	Convert carbohydrates acetic acid and lactic acid	[63]
Sodium benzoate, Sodium nitrite, Potassium sorbate	In vitro	*Clostridium tyrobutyricum or Lactobacillus paracasei*	Anti-inflammatory properties	[64]
Sodium benzoate, Sodium nitrite, Potassium sorbate	In vitro	*Bacteroides thetaiotaomicron or Enterococcus faecalis*	Pro-inflammatory or colitogenic properties	[64]
Benzoic acid	Pig	Phylum of *Bacteroidetes* and the genus of *Prevotella* in the phylum of *Bacteroidetes* ↓	/	[65]
Benzoic acid (90%) and essential oil (10%, include thymol, 2-meth-oxyphenol, and eugenol)	Pig	*Fusicatenibacter*, *Escherichia-Shigella*, and *Ruminococcus* ↑ *Prevotella, and Coprococcus 1* ↓	/	[66]
Potassium sorbate	Mice	*Coriobacteriaceae* ↓	Convert carbohydrates to acetic acid and lactic acid	[63]
Benzoic acid (49%)	Piglet	*Ruminococcus*, *Prevotellaceae*, and *Fibrobacteraceae* ↑	Fiber fermenting abilities	[67]
Nitrite	C57BL/6 mice	*Falsiporphyromonas, Alloprevotella, Acetatifactor, and Coprococcus* ↑ *Akkermansia, and Elusimicrobium* ↓	/	[68]
Sodium benzoate	Human	*Bifidobacterium* ↑	/	[69]
Sodium sulphite	Human	*Escherichia/Shigella* ↑ *Bifidobacterium* ↓	/	[69]
Sulfite	In vitro	*Plantarum*, *rhamnosus*, *Lactobacillus species casei*, and *Streptococcus thermophilus* ↓	/	[70]
NaNO_3_	Mouse	*Lactobacillus, Ruminococcaceae_UCG-014,* and *Prevotellaceae_UCG-001* ↑	/	[71]
Monosodium glutamate (300 mg/kg)	Mice	*Patescibacteria* ↑	/	[72]
Monosodium glutamate (1500 mg/kg)	Mice	*Patescibacteria* ↓	/	[72]
Monosodium glutamate (2 g/day)	Human	No significant difference	/	[73]
Xylitol (10 g/L in high fat diet)	C57BL/6 mice	*Bacteroidetes, Actinobacteria*, and *Proteobacteria* ↓ *Firmicutes* ↑	/	[74]
Xylitol (194 ± 25 mg/kg b.w. in high fat diet)	C57BL/6J mice	*Bacteroidetes, Barnesiella* ↓ *Firmicutes, Prevotella* ↑	/	[75]
Xylitol (10%)	Rat	*Ruminococcaceae*, *Prevotella* ↓ *Bacteroides* ↑	/	[76]
Xylitol	In vitro	*Clostridium, Phascolarctobacterium* ↑	Butyrate synthesizing	[77]
		*Escherichia* ↑	/	
Sorbitol	Mice	*Bifidobacterium, Lachnospiraceae UCG 001, Lachnospiraceae NK4A136, Eubacterium ventriosum, Candidatus Arthromitus, Ruminococcus torques* ↓ *Helicobacter, Tyzzerella, Alistipes, and Prevotella 9* ↑	/	[78]
Sorbitol	C57BL/6 mice	*Escherichia, Klebsiella, Enterobacter, and Proteus in Enterobacteriaceae family*↑	/	[79]
Lactitol	Human	*Actinobacteria, Actinobacteria, Bifidobacteriales, Bifidobacteriaceae and Bifidobacterium* ↑	/	[80]
Titanium dioxide	ICR mice	*Verrucomicrobia, Bacteroidetes* ↓ *Firmicutes* ↑	/	[81]
Sunset yellow	In vitro	*Escherichia coli, Enterococcus faecium, Aerococcus viridans, and Bacillus cereus*	Decolorized sunset yellow	[82]
Resistant starch	Mice	*Lactobacillus, Lachnoclostridium* ↑	/	[49]
Sodium stearoyl lactylate	In vitro	*Clostridia, Clostridiaceae, Lachnospiraceae, Ruminococcaceae* ↓ *Bacteroidaceae and Enterobacteriaceae and Desulfovibrionaceae* ↑	/	[83]

/ means there is no function concerned in the cited literature. ↑ positive influence, ↓ negative influence.

## Data Availability

Not applicable.
